# Evaluation of the role of local therapy in patients with cN1M0 prostate cancer: A population-based study from the SEER database

**DOI:** 10.3389/fonc.2022.1050317

**Published:** 2022-12-05

**Authors:** Feng Qi, Wenbo Xu, Lu Li, Xiao Li, Zicheng Xu

**Affiliations:** ^1^ Department of Urologic Surgery, Jiangsu Cancer Hospital, Jiangsu Institute of Cancer Research, Affiliated Cancer Hospital of Nanjing Medical University, Nanjing, China; ^2^ Department of Urology, Lanzhou University Second Hospital, Lanzhou, China; ^3^ State Key Laboratory of Translational Medicine and Innovative Drug Development, Jiangsu Simcere Diagnostics Co., Ltd., Nanjing, China; ^4^ Department of Scientific Research, Jiangsu Cancer Hospital, Jiangsu Institute of Cancer Research, Affiliated Cancer Hospital of Nanjing Medical University, Nanjing, China

**Keywords:** clinically node-positive, SEER, prostate cancer, local therapy, survival

## Abstract

**Objective:**

To investigate the prognostic value of local therapy (LT) in cN1M0 prostate cancer (PCa).

**Methods:**

Patients diagnosed with cN1M0 PCa were extracted from the surveillance, epidemiology, and end results (SEER) database. Kaplan-Meier (KM) curve was used to compare the survival outcomes between patients treated with and without LT. Further, among patients receiving LT, KM analysis was also applied to investigate the survival differences in patients with radical prostatectomy (RP) and radiation therapy (RT). Propensity score matching (PSM) analysis was performed to balance the basic characteristics of patients in each group and make it comparable when exploring the survival impact of different treatment types. Finally, uni- and multivariable Cox proportional-hazards models were utilized to identify independent prognostic factors associated with overall survival (OS) and cancer-specific survival (CSS) in this population.

**Results:**

Patients treated with LT had significantly better OS (P<0.0001) and CSS (P<0.0001) than those without LT, as well as in most subgroups, except for non-White patients, or those with ISUP grade group 1 or T3 stage. Notably, patients receiving RP also had significantly better OS (P=0.00012) and CSS (P=0.0045) than those treated with RT alone, especially in those aged ≥75 years old, prostate-specific antigen (PSA) 10-20 ng/mL, ISUP grade 1-3 or non-white patients. Finally, clinical T stage, ISUP grade group and the administration of LT were identified to be independent prognostic factors for OS and CSS among cN1M0 PCa patients.

**Conclusion:**

The cN1M0 PCa patients treated with LT were associated with significantly better survival. Among patients receiving LT, the combination of RP and PLND could lead to a better prognosis compared to RT alone in most subgroups. An individualized treatment strategy is warranted to be developed after weighing the benefits and risks of treatment.

## Introduction

Prostate cancer (PCa) is one of the most common malignancies of the male genitourinary system. In 2020, its incidence rate ranked the third among all malignant tumors worldwide (1). Nowadays, more PCa patients have been diagnosed due to the rapid development of imaging technology and the further optimization of prostate biopsy (2, 3). In 2022, the estimated newly diagnosed PCa cases are 268,490 in the United States, accounting for about 27% of all male malignant tumors (4), which has brought serious burden to the male population. Localized PCa can achieve a better prognosis through radical surgery or radiation therapy (RT), and the 5-year overall survival (OS) rate can reach more than 90% (5–7). For advanced diseases, especially for those with metastatic castration-resistant PCa (mCRPC), individualized systemic therapy for different patients is the standard of care. Additionally, various clinical trials are in full swing, new hormone therapy, immunotherapy and targeted therapy (8–11). However, many disputes remained on the treatment of locally advanced diseases.

Previous study reported that approximately 12%-13% of PCa patients were in cN1M0 at the initial diagnosis (positive regional lymph nodes but no distant metastases in images) (12). Moreover, some clinically negative lymph nodes would be confirmed as microscopic nodal involvement after extended pelvic lymph node dissection (ePLND) (13). However, PCa patients will be grouped into IV stage once there are positive lymph node (with or without the confirmation by lymphadenectomy or biopsy) in clinical evaluation, regardless of the prostate-specific antigen (PSA) value, clinical T stage or International Society of Urological Pathology (ISUP) grade group (14), implying the incurability of tumor itself. Many scholars believed that the disease has surpassed the prostate itself, therefore, local therapy (LT) was unnecessary or far from enough due to the limited survival benefits. Accordingly, standard care of these patients is mostly based on androgen deprivation therapy (ADT) combined with non-curative treatment.

Previous studies (15–17) have explored the potential prognostic value of LT in cN1M0 PCa patients, most of them supported the combination of ADT with LT (primarily RT). However, the value of radical prostatectomy (RP) addition to ADT in cN1M0 PCa patients is still controversial, and recommendations for RP in major clinical guidelines are also inconsistent. In European Association of Urology (EAU) guidance, systemic treatment combined with LT in locally advanced patients could provide the best outcome. Among them, RT and RP are both recommended as potential alternatives for LT. However, RP is not recommended for the treatment of cN1M0 PCa patients in the National Comprehensive Cancer Network (NCCN) clinical guidance.

In addition, no randomized controlled and prospective studies have compared the prognostic value of RT and RP in cN1M0 PCa patients. Seisen et al. (18) explored the effect of LT ± ADT versus ADT alone in cN1 patients. They found that no significant differences were detected in the comparison between RP ± ADT and RT ± ADT in overall mortality-free survival [hazard ratio (HR)=0.54, 95% confidence interval (CI)=0.19-1.52, P=0.2]. Similarly, Sarkar et al. (17) demonstrated that no statistically differences were detected in cancer-specific mortality (CSM) or all-cause mortality (ACM) between RP and RT in patients with cN1 PCa. In these two studies, RP favored a survival benefit over RT although there was no statistical difference.

Therefore, we carried out this study to compare the prognostic value of LT for cN1M0 diseases in the propensity score matching (PSM) population, and screened for specific populations that could potentially benefit from LT. Subsequently, we compared the value of RT versus the combined use of RP and PLND in OS and cancer-specific survival (CSS). Finally, independent risk factors involved in the prognosis were identified by multivariable Cox regression models.

## Methods and materials

### Database

All the raw data of this study were retrospectively collected from the surveillance, epidemiology, and end results (SEER) database. SEER database is a public database covering about 34.6% of the U.S. population (https://surveillance.cancer.gov/statistics/types/race_ethnic.html) and is designed to collect and publish the incidence rate, basic characteristics, mortality and long-term survival outcomes of cancer patients. Before accessing the database, we carefully read and signed the Data Agreement. This study was exempt by Institutional Review Board (IRB) approval because all the data involved in this study were from this open assess database. Moreover, SEER Registry also has some other extended databases, including SEER-Medicare, SEER-MHOS, and SEER-CAHPS. Researchers can select appropriate databases according to specific requirements.

### Identification of cN1M0 PCa patients

**Figure 1:**
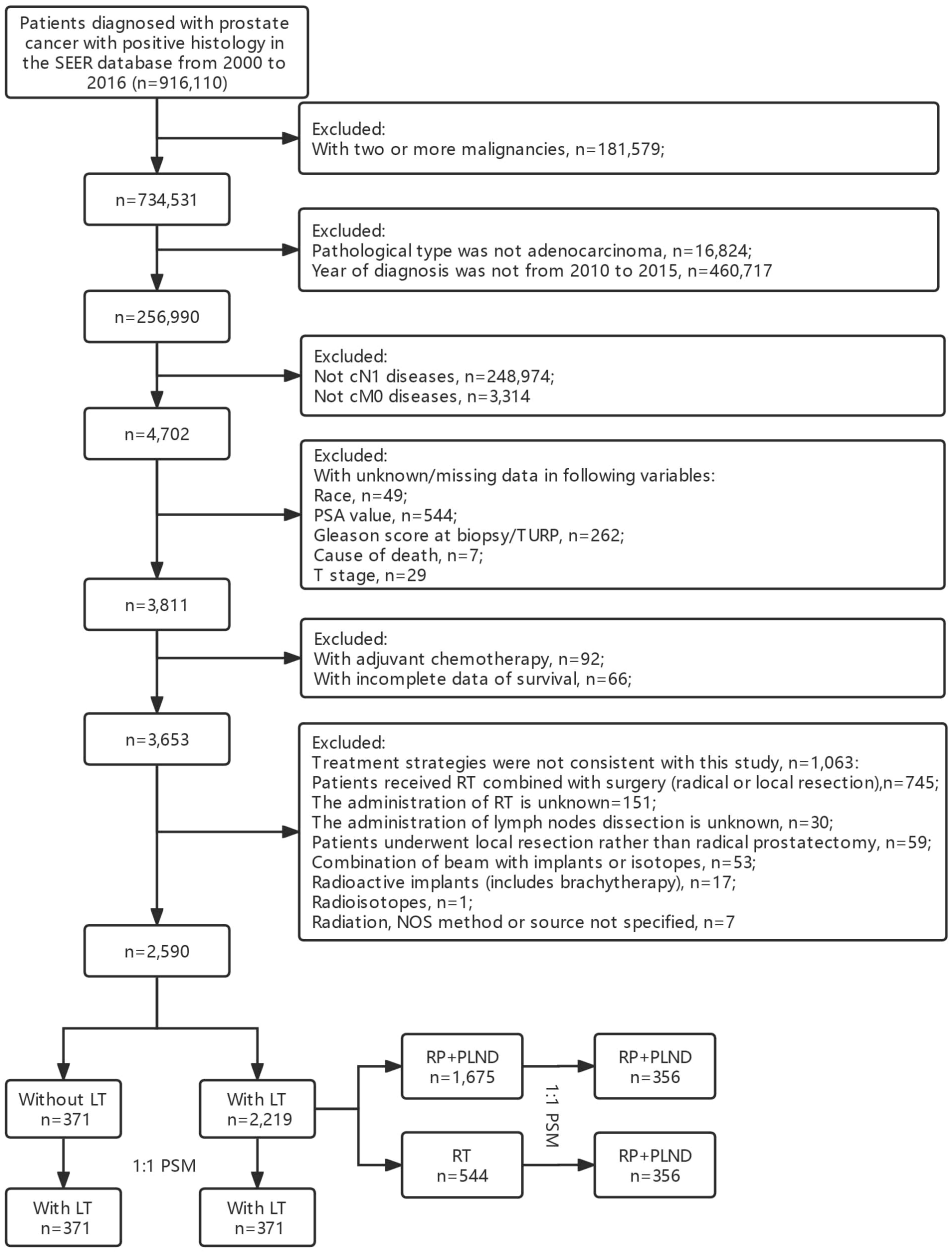
Flowchart of patient selection.

The main purpose of this study was to investigate the prognostic role of LT in cN1M0 PCa patients. We used the “Case Listing Session” tool to extract patients diagnosed with cN1M0 PCa between 2010 and 2015 from the SEER database. The inclusion criteria were as follows: (1) patients were confirmed as PCa with positive histology [C74.9, International Classification of Disease for Oncology (ICD-O): 8140/3]; (2) patients with positive lymph nodes in clinical evaluation, but no distant metastasis (cTanyN1M0); (3) year of diagnosis was between 2010 and 2015. Additionally, the exclusion criteria were as follows: (1) PCa was not the only malignancy of each patient; (2) pathological type was not adenocarcinoma; (3) patients with missing or unknown data in some important variables, including race, prostate-specific antigen (PSA) value, Gleason score at biopsy or transurethral resection of prostate (TURP), TNM stage, vital status, cause of death and survival time; (4) patients with adjuvant/neoadjuvant chemotherapy; (5) combined therapy strategies were not consistent with our study; (6) reporting source was from autopsy/death certificate only. The selection flowchart was in **Figure 1**.

### Data extraction and processing

We extracted the basic characteristics, pathological results and long-term survival outcomes of enrolled patients, including age at diagnosis, race recode, year of diagnosis, TNM stage, the administration of RP, PLND and beam radiation, PSA value, Gleason score, vital status, survival months, cause of death, household income and so on.

Race was grouped into White, Black and Other (including American Indian/AK Native, Asian/Pacific Islander). Clinical T stage was grouped into four groups (T1-4) according to the variable “CS Extension-Clinical Extension”. Furthermore, the variable “RX Summ–Scope Reg LN Sur (2003+)” was used to determine whether patients underwent regional lymph nodes dissection. Only patients with regional lymph nodes removed would be further analyzed, and those with biopsy or aspiration only would be excluded. RT for the primary site is specifically referred to external beam radiation therapy (EBRT), not brachytherapy.

Patients were divided into two groups (with vs. without LT) based on whether they had local treatment for the primary tumor. Moreover, patients treated with LT were further divided into two groups for subsequent analysis: RP + PLND vs. RT. Propensity score matching (PSM) analysis was performed with a ratio of 1:1 by the nearest neighbor matching method to balance the basic characteristics of patients in each group and make it comparable when exploring the survival impact of different treatment types, variables in PSM analysis included age, race, clinical T stage, PSA, ISUP grade group, and household income.

### Survival outcomes

In total patients, survival differences between patients treated with or without LT was explored by Kaplan–Meier (KM) analyses both before and after PSM. Additionally, Cox proportional-hazards model was used to evaluate the prognostic role of LT in each subgroup. Then, in patients treated with LT, KM analyses were developed to investigate the survival differences caused by two definitive treatment strategies (RP+PLND versus RT), as well as in different subgroups. Finally, uni- and multivariate Cox regression models were utilized to investigate independent risk factors for OS and CSS in cN1M0 PCa patients.

### Statistical analyses

In this study, continuous variables that do not conform to the normal distribution were displayed in the form of median [(interquartile range), IQR] and compared using Mann Whitney U test. Categorical variables were presented in the form of n (%) and compared with Fisher’s exact test or Chi-square test. PSM was developed for balancing the basic characteristics between different groups. KM and Cox survival analyses were constructed to explore the prognosis of different treatment methods in cN1M0 PCa patient. Finally, uni- and multivariate Cox regression models were utilized to investigate independent risk factors for OS and CSS in cN1M0 PCa patients. In this study, statistical analyses were produced by SPSS 23.0 (SPSS Inc, Chicago, IL, USA) and R (V3.4.1). A two-sided *P*<0.05 was statistically significant.

## Results

### Basic characteristics

A total of 2,590 cN1M0 PCa patients were enrolled in this study, of which 85.68% (2,219/2,590) received LT, while 14.32% (371/2,590) of the patients had no definitive LT for primary tumor. As shown in **Table S1**, the median (IQR) age at diagnosis was 64 (59-69) years and the median (IQR) PSA was 11.95 (7.0-23.7) ng/mL. Most patients were White (80.85%), with earlier T stage (T1-2: 79.61%). In addition, the Gleason scores at biopsy or TUPR were mostly 7 (34.71%) and 8 (56.91%). Patients treated with LT had younger age at diagnosis (median: 64 vs. 68 years old, P<0.001), lower PSA value (median: 11.00 vs. 19.80 ng/mL, P<0.001), lower T stage (T1: 45.83% vs. 39.08%; T2: 35.20% vs. 32.08%, P<0.001) and lower ISUP grade group (ISUP 1: 4.24% vs. 3.77%; ISUP 2: 37.58% vs. 17.52%, P<0.001) when compared with those without LT. No significant difference was detected in racial distribution between two groups (P=0.175). Therefore, PSM analysis was performed to eliminate the difference in survival between the two groups due to mismatches in basic features. Comparisons between patients treated with and without LT in basic characteristics showed no significant differences (**Table S2**).

In patients treated with LT (**Table S3**), 75.48% (1,675/2,219) of the patients received RP+PLND, while 24.52% (544/2,219) of the patients treated with RT only. Patients treated with RP+PLND had younger age at diagnosis (median: 63 vs. 67 years old, P<0.001), lower PSA value (median: 10.2 vs. 17.6 ng/mL, P<0.001), lower T stage (T1: 51.1% vs. 29.6%; T2: 36.78% vs. 30.33%, P<0.001), lower ISUP grade group (ISUP 1: 4.90% vs. 2.21%; ISUP 2: 43.16% vs. 20.40%, P<0.001) and lower median household income (low level: 62.39% vs. 48.90%, P<0.001). Similarly, no significant differences were detected in the comparisons of basic characteristics between patients treated with RT and RP+PLND (**Table S4**).

### Survival outcomes

**Figure 2:**
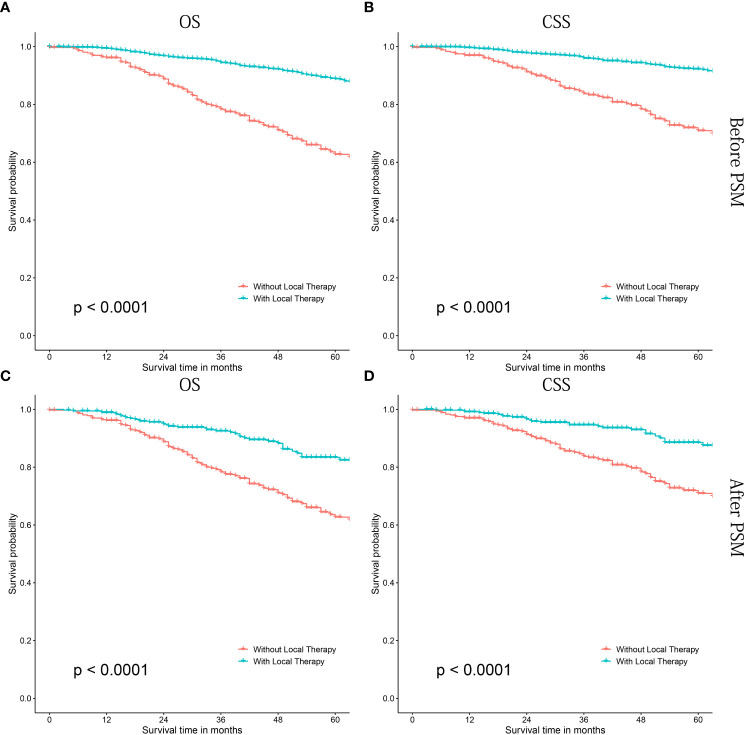
Kaplan-Meier survival curves of cN1M0 prostate cancer patients treated with or without local therapy. **(A, B)** OS and CSS before PSM; **(C, D)** OS and CSS after PSM. OS, overall survival; CSS, cancer-specific survival; PSM, propensity score matching.

As shown in **Figure 2**, patients treated with LT had significant better OS (P<0.0001, **Figure 2A**) and CSS (P<0.0001, **Figure 2B**) than those without LT. Similarly, significant survival benefits were found in patients treated with LT after PSM (P<0.0001 for OS and CSS) (**Figures 2C, D**). In addition, we performed a COX proportional regression model to investigate whether LT conferred a survival benefit in all subgroups.

**Figure 3:**
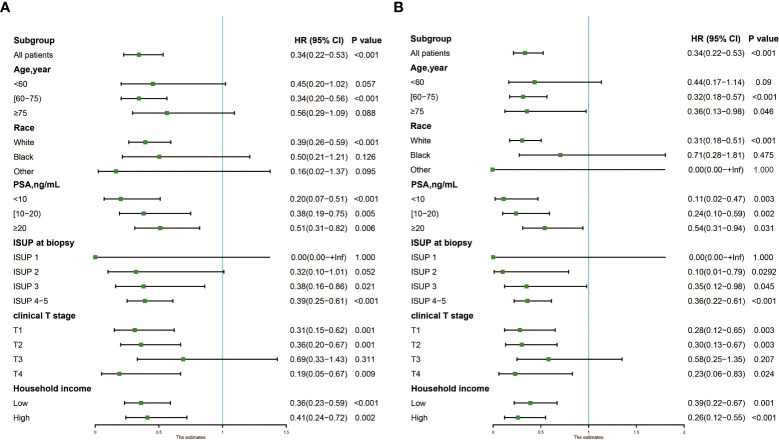
Forest plot showing results of Cox regression model for evaluating the prognostic role of local therapy in each subgroup. **(A)** OS; **(B)** CSS. OS, overall survival; CSS, cancer-specific survival.

The forest plot (**Figure 3**) showed that patients treated with LT could confer significant OS (**Figure 3A**) and CSS (**Figure 3B**) benefits in most subgroups, except for non-White patients [Black: P=0.126 and 0.475 for OS and CSS; Other: P=0.095 and 1.000 for OS and CSS], or those with ISUP grade group 1 (P= 1.000 for OS and CSS) or T3 stage (P=0.311 and 0.207 for OS and CSS). In some subgroups, although the survival benefits of LT were not statistically significant, there was still a significant trend.

**Figure 4:**
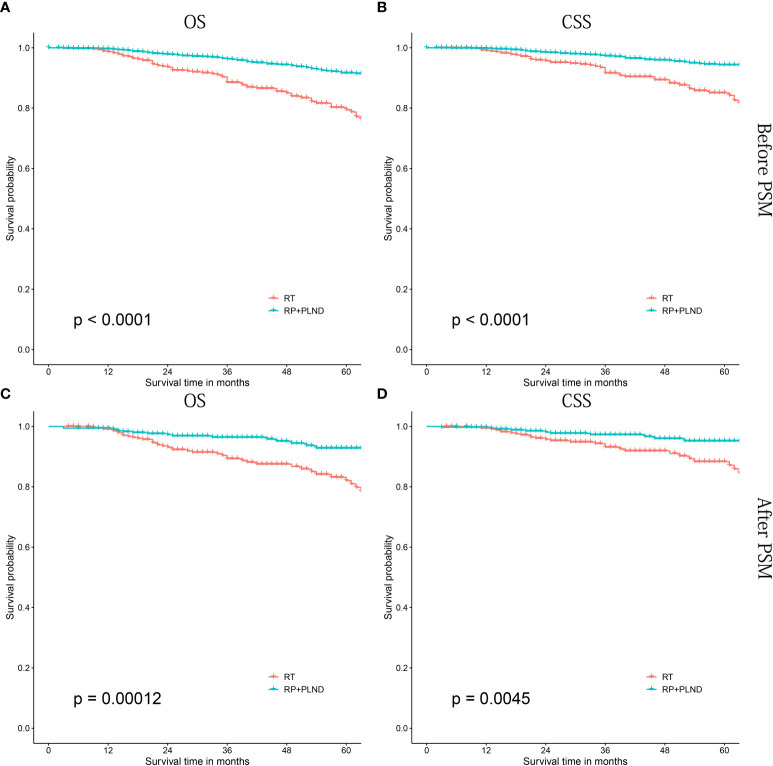
Kaplan-Meier survival curves of cN1M0 prostate cancer patients treated with RP+PLND versus radiation therapy. **(A, B)** OS and CSS before PSM; **(C, D)** OS and CSS after PSM. RP, radical prostatectomy; PLND, pelvic lymph node dissection; OS, overall survival; CSS, cancer-specific survival; PSM, propensity score matching.

In patients treated with LT, patients receiving RP+PLND had significantly better OS and CSS than those treated with RT alone (both before [OS: P<0.0001; CSS: P<0.0001] and after PSM [OS: P=0.00012; CSS: P=0.0045], **Figures 4A–D**). Then, further survival analyses were performed to investigate the differences between RP+PLND and RT in different subgroups stratified by age at diagnosis (**Figures S1**, **S2A–C**), PSA value (**Figures S1**, **S2D–F**), ISUP grade group (**Figures S1**, **S2G–I**), clinical T stage (**Figures S1**, **S2J-L**), household income (**Figures S3**, **S4A, B**) and race (**Figures S3**, **S4C-E**).

RP+PLND seemed to be a better choice than RT, but for some specific subgroups, patients receiving RT had similar long-term prognosis (both OS and CSS) than that of patients receiving RP+PLND, especially in those aged ≥75 years old, PSA 10-20 ng/mL, ISUP grade group 1-3 or non-white. In addition, RP+PLND and RT were comparable in CSS in patients with clinical T1-2 stage, while patients treated with RP had better OS (cT1: P=0.014; cT2, P=0.056 critical).

### Uni- and multivariate Cox regression model

In cN1M0 PCa patients, potential prognostic risk factors were initially screened out by univariate Cox regression model. Then, variables with statistical significance (P<0.05) were further included in multivariate Cox analyses to identify independent risk factors affecting the prognosis of patients in this population. Univariate Cox analyses found that younger age at diagnosis, lower tumor grade, lower clinical T stage, lower PSA, lower ISUP grade group and the administration of LT may be associated with better OS and CSS (**Tables 1**, **2**). Finally, multivariate Cox analyses showed that clinical T stage (OS: P=0.018; CSS: P=0.027), ISUP grade group (OS: P<0.001; CSS: P<0.001) and the administration of LT (OS: P<0.001; CSS: P<0.001) were identified to be independent risk factors for OS and CSS in cN1M0 PCa patients.

**Table 1:** Uni- and multivariate Cox regression models for predicting OS in cN1M0 prostate cancer patients.

Variable	Univariate			Multivariate		
	HR	95% CI	P value	HR	95% CI	P value
Age			**<0.001**			0.552
<60	Reference			Reference		
[60,75)	1.117	0.832-1.500	0.462	0.955	0.709-1.286	0.762
>75	2.458	1.702-3.550	<0.001	1.147	0.780-1.686	0.487
Race			0.927			
White	Reference			Reference		
Black	0.951	0.668-1.354	0.781			
Other	1.073	0.613-1.879	0.805			
Clinical T stage			**<0.001**			**0.018**
T1	Reference			Reference		
T2	1.227	0.918-1.640	0.166	1.074	0.801-1.440	0.633
T3	1.697	1.213-2.374	0.002	1.154	0.815-1.633	0.421
T4	4.747	3.045-7.400	<0.001	2.098	1.316-3.346	0.002
PSA			**<0.001**			0.214
<4	Reference			Reference		
[4-10)	0.596	0.340-1.046	0.071	0.719	0.408-1.268	0.254
[10-20)	0.956	0.549-1.664	0.873	0.944	0.538-1.654	0.839
>20	1.339	0.780-2.297	0.290	1.002	0.579-1.735	0.994
ISUP			**<0.001**			**<0.001**
ISUP 1	Reference			Reference		
ISUP 2	1.075	0.457-2.526	0.869	1.073	0.456-2.527	0.872
ISUP 3	1.814	0.779-4.225	0.167	1.523	0.651-3.566	0.332
ISUP 4	3.755	1.655-8.521	0.002	2.702	1.184-6.170	0.018
ISUP 5	8.467	3.559-20.141	<0.001	4.116	1.698-9.978	0.002
Household income			0.843			
Low	Reference					
High	0.976	0.764-1.247	0.843			
Local Therapy			**<0.001**			**<0.001**
No	Reference			Reference		
RT	0.510	0.377-0.690	<0.001	0.523	0.383-0.714	<0.001
RP+PLND	0.184	0.139-0.245	<0.001	0.288	0.209-0.395	<0.001

OS, overall survival; ISUP, International Society of Urological Pathology; RT, radiotherapy; RP, radical prostatectomy; PLND, pelvic lymph node dissection HR, hazard ratio; CI, confidence interval. Bold values represent statistically significant.

**Table 2:** Uni- and multivariate Cox regression models for predicting CSS in cN1M0 prostate cancer patients.

Variable	Univariate			Multivariate		
	HR	95% CI	P value	HR	95% CI	P value
Age			**0.007**			0.490
<60	Reference			Reference		
[60,75)	0.990	0.707-1.387	0.953	0.830	0.591-1.168	0.285
>75	1.850	1.181-2.896	0.007	0.786	0.491-1.259	0.316
Race			0.982			
White	Reference					
Black	1.016	0.675-1.531	0.938			
Other	0.941	0.462-1.916	0.866			
Clinical T stage			**<0.001**			**0.027**
T1	Reference			Reference		
T2	0.979	0.688-1.395	0.908	0.835	0.584-1.195	0.325
T3	1.718	1.169-2.524	0.006	1.140	0.764-1.701	0.523
T4	4.769	2.865-7.938	<0.001	1.905	1.111-3.265	0.019
PSA			**<0.001**			0.187
<4	Reference			Reference		
[4-10)	0.532	0.274-1.034	0.063	0.620	0.317-1.214	0.163
[10-20)	0.921	0.481-1.764	0.804	0.826	0.426-1.601	0.571
>20	1.320	0.702-2.482	0.388	0.938	0.493-1.785	0.845
ISUP			**<0.001**			**<0.001**
ISUP 1	Reference			Reference		
ISUP 2	0.977	0.341-2.802	0.966	0.984	0.342-2.828	0.976
ISUP 3	1.892	0.673-5.317	0.277	1.676	0.593-4.741	0.330
ISUP 4	3.829	1.404-10.441	0.009	2.877	1.047-7.907	0.041
ISUP 5	11.988	4.244-33.863	<0.001	6.432	2.224-18.595	0.001
Household income			0.606			
Low	Reference					
High	0.926	0.690-1.242	0.606			
Local Therapy			**<0.001**			**<0.001**
No	Reference			Reference		
RT	0.479	0.335-0.686	<0.001	0.480	0.331-0.695	<0.001
RP+PLND	0.167	0.119-0.234	<0.001	0.273	0.187-0.399	<0.001

CSS, cancer specific survival; ISUP, International Society of Urological Pathology; RT, radiotherapy; RP, radical prostatectomy; PLND, pelvic lymph node dissection HR, hazard ratio; CI, confidence interval. Bold values represent statistically significant.

## Discussion

In the 8th edition of American Joint Committee on Cancer (AJCC) staging manual, PCa patients with positive lymph nodes were divided into stage IV A. Therefore, the value of definitive therapy in this population was minimized because many investigators believed that cN1 PCa represented a state of systemic disease with a high risk of metastasis (19). Moreover, being grouped into the same stage (stage IV) with M1 patients can also cause unnecessary anxiety for patients and their caregivers (20). Shinde et al. (20) reposted that approximately one-third of cN1M0 patients did not received any definitive therapy for their disease.

Several previous studies have explored the prognostic value of LT in this population. However, conclusions were still controversial, and the specific beneficiary population was still fuzzy. In addition, there were no special requirements for the selection of LT. Should we choose RT or RP? Is non-invasive RT an alternative to RP+PLND in some patients? What are the independent risk factors affecting the prognosis of this patients, and how to balance the treatment risks and survival benefits? Moreover, the decrease in quality of life caused by LT, especially radical surgery, is also an important factor to be weighed. Therefore, we conducted this study to address the above issues.

In our study, compared patients receiving non-definitive therapy, those treated with LT (RT or RP+PLND) had significantly better survival outcomes than those without. Sarkar et al. (17) investigated the role of RP in clinically node-positive PCa patients, they found that RP was tightly related to significantly better prostate CSM (subdistribution HR: 0.32, 95% CI: 0.16–0.66; P = 0.002) and ACM (HR: 0.36, 95% CI 0.21–0.61; P< 0.001). Tward et al. (21) explored the effect of RT for clinically node-positive prostate adenocarcinoma on survival outcomes. They concluded that RT was associated with improved OS and CSS than non-definitive therapy. Similarly, Lin et al. (22) demonstrated that cN+ patients treated with ADT+RT had significantly better survival outcomes than those receiving ADT alone, and ADT+RT was related to a 50% decreased risk of 5-year ACM.

The above studies have confirmed the prognostic value of LT in cN1M0 PCa patients, however, are there some subgroups in which LT is insignificant or unnecessary? Bryant et al. (23) compared the survival outcomes between cN1M0 PCa patients treated with ADT+RT and ADT alone. They found that ADT+RT was associated with better CSS and OS than ADT alone in patients with PSA levels less than 26 ng/mL, while no significant survival differences were detected in patients with PSA levels higher than 26 ng/mL. Leiri et al. (15) explored the candidates for LT with RT in clinically node-positive PCa patients. They found that patients with high-risk features (at least two of the three among ≥75% biopsy positive core rate, ISUP group grade 5, and ≥2 positive lymph nodes) were more likely to benefit from RT.

In our study, we found that the comparison between LT and non-LT was not statistically different in non-White patients, or those with ISUP grade group 1 or T3 stage. ISUP grade group 1, defined as Gleason score 3 + 3, is usually considered to be clinically insignificant PCa. If clinical N stage is not considered, such tumors often present with an indolent state. Therefore, many patients prefer conservative treatment to RT or invasive surgery. In EAU clinical guidance, active surveillance is strongly recommended in patients with Gleason score 3 + 3. Additionally, imaging techniques with highly sensitive could result in a stage shift with more patients classified as node-positive. In fact, many lymph nodes would be confirmed as inflamed or hyperplastic lymph nodes; even if with microscopic nodal involvement, the burden is very low. The above may be the reason why the prognosis in patients with ISUP grade group 1 but cN1 who received non definitive therapy was not inferior to that in patients who received LT. Non-White patients treated with LT had similar survival outcomes than those without LT (both CSS and OS). PCa has always been considered as a tumor with great racial heterogeneity (24, 25). However, studies on ethnic differences in the prognostic value of LT in cN1 patients are largely absent. Obviously, racial differences in cancer cannot be simply attributed to environmental or geographical factors, but should also be explored at the micro level. An immunosuppressive tumor microenvironment caused by allelic variants and hyper-expression of genes in African Americans may result in more aggressive tumors and worse prognosis than White patients (26). Interestingly, no survival differences were detected between cT3 patients treated with and without LT, while significant survival benefits were found in T1-2 and T4 PCa patients. It seems very difficult to explain why this difference in survival occurs in such an intermediate stage of PCa patients. Further studies are still needed to verify the conclusions of this study, especially in the molecular mechanism.

RT has been well studied as an alternative of LT and proved to have significant prognostic value in cN1 patients. However, there is no sufficient evidence to support the prognostic value of RP in these patients, and its recommendations in EAU and NCCN guidelines are on the contrary. Chierigo et al. (27) investigated the prognosis of RP versus RT in clinical node-positive PCa patients, and they found that the 5-year ACM and CSM rates were 15.4% versus 25% and 9.3% versus 17% for RP versus RT (all P<0.05). However, they only detected the survival advantage of RP in the general population without further subgroup analyses. Moreover, they did not state the administration of PLND, and it seemed unreasonable to ignore the critical role of regional lymph nodes dissection in PCa patients with clinically suspected positive lymph nodes. Furthermore, Sarkar et al. (17) found that RP had comparable survival outcomes when compared with RT. However, they did not match the basic characteristics of patients in the two groups, and the difference in baseline may lead to large selection bias. In addition, they did not elaborate on PLND.

In our study, patients treated with RP+PLND had significantly better prognosis (OS and CSS) than those receiving EBRT. Moreover, RP seemed to be a better choice in most subgroups when stratified by clinical variables. However, non-invasive RT could lead to comparable survival outcome as RP, especially in patients aged ≥75 years old, PSA 10-20 ng/mL, ISUP grade group 1-3 or non-White patients. In addition, in patients with clinical T1-2, comparable in CSS was found in patients treated with RT and RP+PLND. Therefore, although RP+PLND had more advantages in the treatment of cN1M0 patients, non-invasive RT can also be an alternative in specific populations. Finally, clinical T stage, ISUP grade group and the administration of LT were identified to be independent risk factors for OS and CSS in cN1M0 PCa patients, this also confirmed the value of LT in cN1M0 PCa patients. Moreover, the HRs of RP versus RT were 0.552 (95% CI=0.390-0.780, P=0.001) for OS and 0.572 (95% CI=0.375-0.872, P=0.009) for CSS (data were not shown), which further highlighted the survival advantages of RP+PLND. However, compared to RP+PLND, non-invasive RT had similar survival outcomes in selected patients. Therefore, clinicians should balance the risks and benefits of invasive treatment when formulating treatment strategies.

Although ePLND is the gold standard for lymph node staging, lymph node metastasis may be missed by ePLND or misclassified by histology. Nowadays, new imaging technologies have developed rapidly. Prostate-specific membrane antigen (PSMA) PET-CT has become an important tool for early diagnosis, accurate staging and recurrence evaluation of PCa. Luiting et al. (28) demonstrated that the sensitivity and specificity of gallium-68 (68 Ga)-PSMA PET for detecting pelvic lymph node metastases in patients with primary PCa were 33.3% to 100% and 80% to 100%, respectively. In another study, Hope et al. (29) evaluated the accuracy of 68Ga-PSMA PET imaging for detecting pelvic nodal metastases compared with histopathology, and the sensitivity and specificity were 0.40 (95% CI, 0.34-0.46) and 0.95 (95% CI, 0.92-0.97) respectively. It was reported that lymph node metastases would be missed in 0-9% of primary prostate tumors and lymph node metastases due to the lack of PSMA expression (30, 31). In addition, 68Ga-PSMA PET would miss lymph node metastases smaller than 5mm (32–34). Hence, imaging evaluation has high specificity for detecting lymph node metastasis, but its sensitivity is moderate. In clinical practice, we need to comprehensively assess the risk of lymph node metastasis and develop personalized treatment strategies for each patient.

However, there were some limitations that cannot be ignored. The SEER data lacks the information about the administration of ADT, which is crucial for the management of PCa patients. With the emergence of some new treatments (abiraterone, enzalutamide, apalutamide and darolutamide), patients from 2010 to 2015 in the SEER database might nor well-represent the current situation. In addition, we can only obtain the number of dissected lymph nodes from the SEER database, but not the extent of dissection, as well as the RT field (extent and Gy). Furthermore, we can only obtain the status of lymph nodes (clinically positive or negative), but not the evaluation manner (CT scan or MRI) and the dimensional cutoff in cm. Obviously, the evaluation method is also an important factor affecting clinical judgment especially in the era of new imaging technologies PSMA PET-CT. Moreover, some important variables were missing or blank, such as prostate imaging reporting and data system (PI-RADS) score, complications and so on. LT might be a proxy of overall higher intensity of treatment, and patients who did not receive it might have received an overall suboptimal treatment. Finally, although a PMS analysis has been performed, selection bias was unavailable due to the retrospective non randomized nature. Therefore, further prospective and well-studied studies are needed in the future to validate our results.

## Conclusion

In cN1M0 PCa diseases, patients who received LT were associated with significantly better survival outcomes. Compared with RT, RP+PLND could lead to a better prognosis in most patients. However, in some specific populations, RT and RP+PLND had comparable survival outcomes. Therefore, an individualized treatment strategy should be developed after weighing the benefits and risks of treatment.

## Data availability statement

The original contributions presented in the study are included in the article/**Supplementary Material**. Further inquiries can be directed to the corresponding authors.

## Ethics statement

All SEER data were accessed with approval from the SEER database, and, as such, this article does not contain any studies with human participants or animals performed by any of the authors. Research on the de-identified data from the SEER program was exempt from the need for institutional review board (IRB) approval by convention, and informed consent was not required. All procedures performed in human subjects studies were conducted in conformity with the Helsinki declaration of 1964 and its subsequent amendments or comparable ethical standards.

## Author contributions

ZX, FQ, WX, and XL conceived and designed the analysis, collected the data, performed the analysis, and wrote the paper. LL and FQ conceived and designed the analysis, contributed data or analysis tools, performed the analysis. FQ and WX interpreted the data and wrote the paper. ZX and LL conceived and designed the analysis and interpreted the data. All authors contributed to the article and approved the submitted version.

## Funding

This work was supported by the Research Project of Jiangsu Cancer Hospital (ZM202015).

## Acknowledgments

The authors sincerely acknowledge Prof Meilin Wang (Department of Environmental Genomics, Jiangsu Key Laboratory of Cancer Biomarkers, Prevention and Treatment, Collaborative Innovation Center for Cancer Personalized Medicine, Nanjing Medical University) for his contributions to the study.

## Conflict of interest

Author LL was employed by Jiangsu Simcere Diagnostics Co., Ltd. and Nanjing Simcere Medical Laboratory Science Co., Ltd.

The remaining authors declare that the research was conducted in the absence of any commercial or financial relationships that could be construed as a potential conflict of interest.

## Publisher’s note

All claims expressed in this article are solely those of the authors and do not necessarily represent those of their affiliated organizations, or those of the publisher, the editors and the reviewers. Any product that may be evaluated in this article, or claim that may be made by its manufacturer, is not guaranteed or endorsed by the publisher.

## Supplementary material

Supplementary Figure 1 Kaplan-Meier curves of OS for cN1M0 prostate cancer patients treated with RP+PLND versus radiation therapy, stratified by different variables: age at diagnosis **(A–C)**, PSA **(D–F)**, ISUP grade group **(G–I)** and clinical T stage **(J–L)**. OS, overall survival; RP, radical prostatectomy; PLND, pelvic lymph node dissection; PSA, prostate-specific antigen; ISUP, International Society of Urological Pathology.

Supplementary Figure 2 Kaplan-Meier curves of CSS for cN1M0 prostate cancer patients treated with RP+PLND versus radiation therapy, stratified by different variables: age at diagnosis **(A–C)**, PSA **(D–F)**, ISUP grade group **(G–I)** and clinical T stage **(J–L)**. CSS, cancer-specific survival; RP, radical prostatectomy; PLND, pelvic lymph node dissection; PSA, prostate-specific antigen; ISUP, International Society of Urological Pathology.

Supplementary Figure 3 Kaplan-Meier curves of OS for cN1M0 prostate cancer patients treated with RP+PLND versus radiation therapy, stratified by different variables: household income **(A, B)** and race **(C–E)**. OS, overall survival; RP, radical prostatectomy; PLND, pelvic lymph node dissection.

Supplementary Figure 4 Kaplan-Meier curves of CSS for cN1M0 prostate cancer patients treated with RP+PLND versus radiation therapy, stratified by different variables: household income **(A, B)** and race **(C–E)**. CSS, cancer-specific survival; RP, radical prostatectomy; PLND, pelvic lymph node dissection.
