# Supplementary figures and images for: Evaluation of the role of local therapy in patients with cN1M0 prostate cancer: A population-based study from the SEER database
Source: Front Oncol. 2022 Dec 5;12:1050317. doi: 10.3389/fonc.2022.1050317 (PMC9760928; doi:10.3389/fonc.2022.1050317)

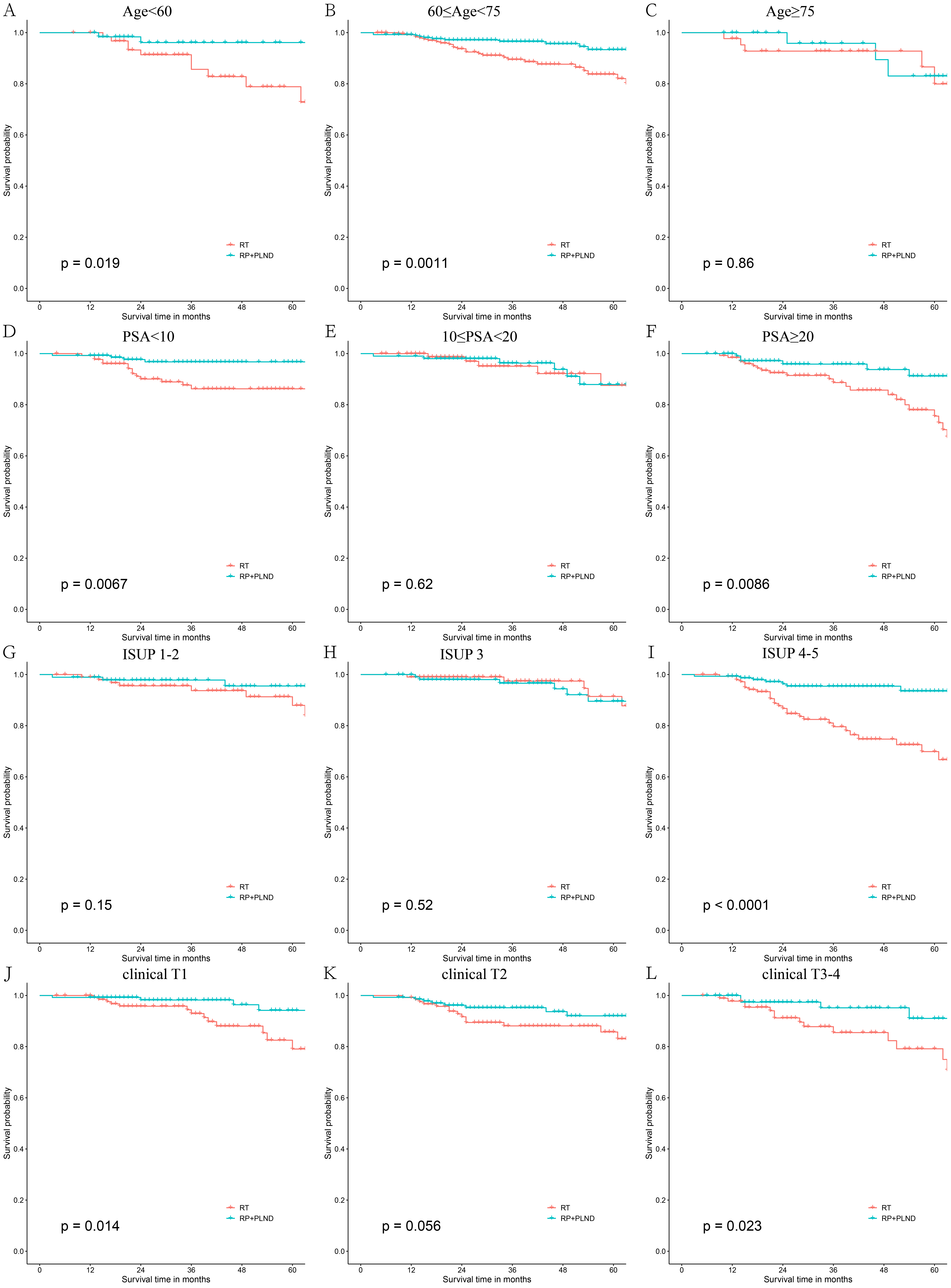

Supplement: Supplementary Figure 1 — Kaplan-Meier curves of OS for cN1M0 prostate cancer patients treated with RP+PLND versus radiation therapy, stratified by different variables: age at diagnosis (A–C), PSA (D–F), ISUP grade group (G–I) and clinical T stage (J–L). OS, overall survival; RP, radical prostatectomy; PLND, pelvic lymph node dissection; PSA, prostate-specific antigen; ISUP, International Society of Urological Pathology. [file Image_1.jpeg]

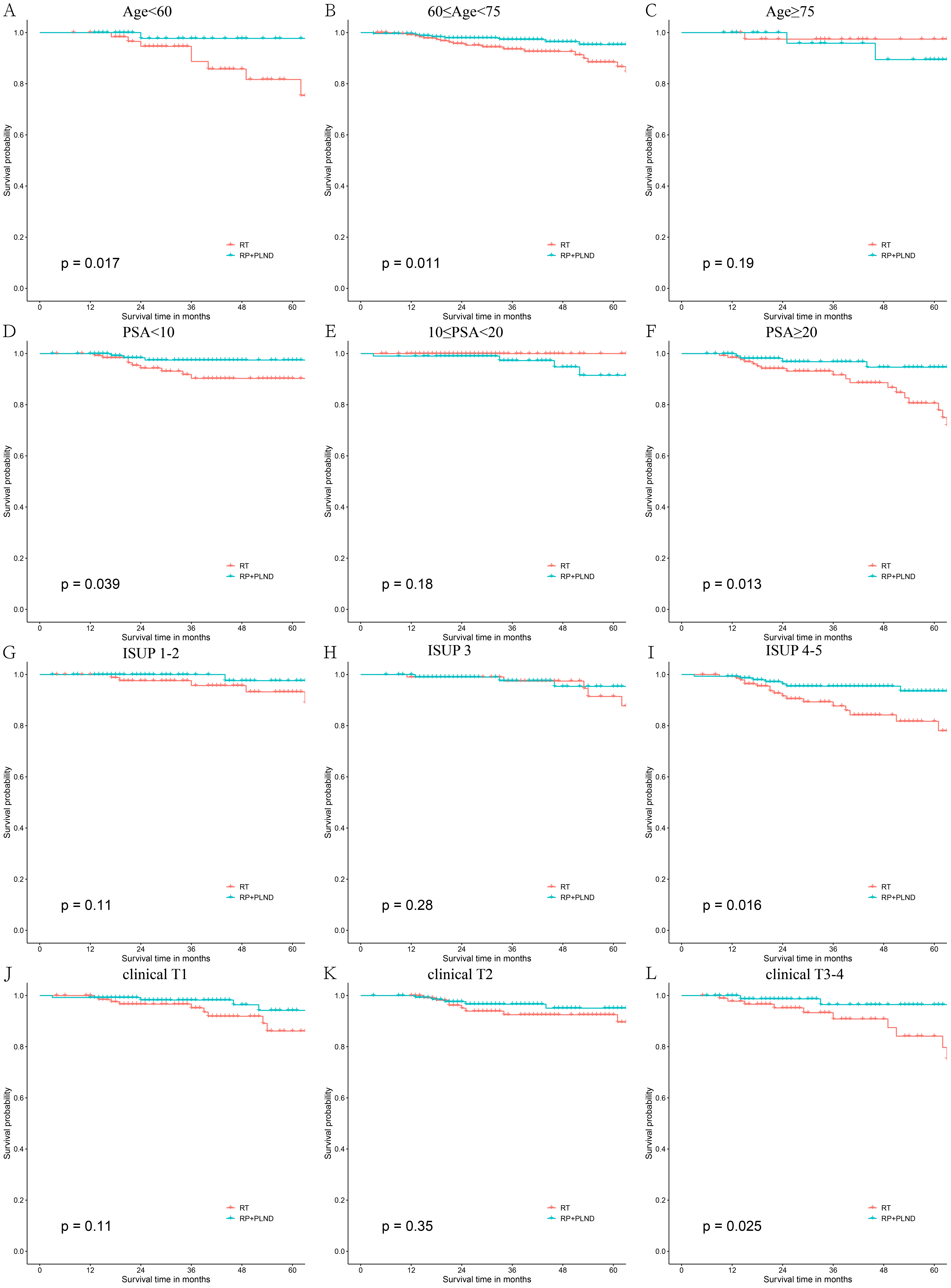

Supplement: Supplementary Figure 2 — Kaplan-Meier curves of CSS for cN1M0 prostate cancer patients treated with RP+PLND versus radiation therapy, stratified by different variables: age at diagnosis (A–C), PSA (D–F), ISUP grade group (G–I) and clinical T stage (J–L). CSS, cancer-specific survival; RP, radical prostatectomy; PLND, pelvic lymph node dissection; PSA, prostate-specific antigen; ISUP, International Society of Urological Pathology. [file Image_2.jpeg]

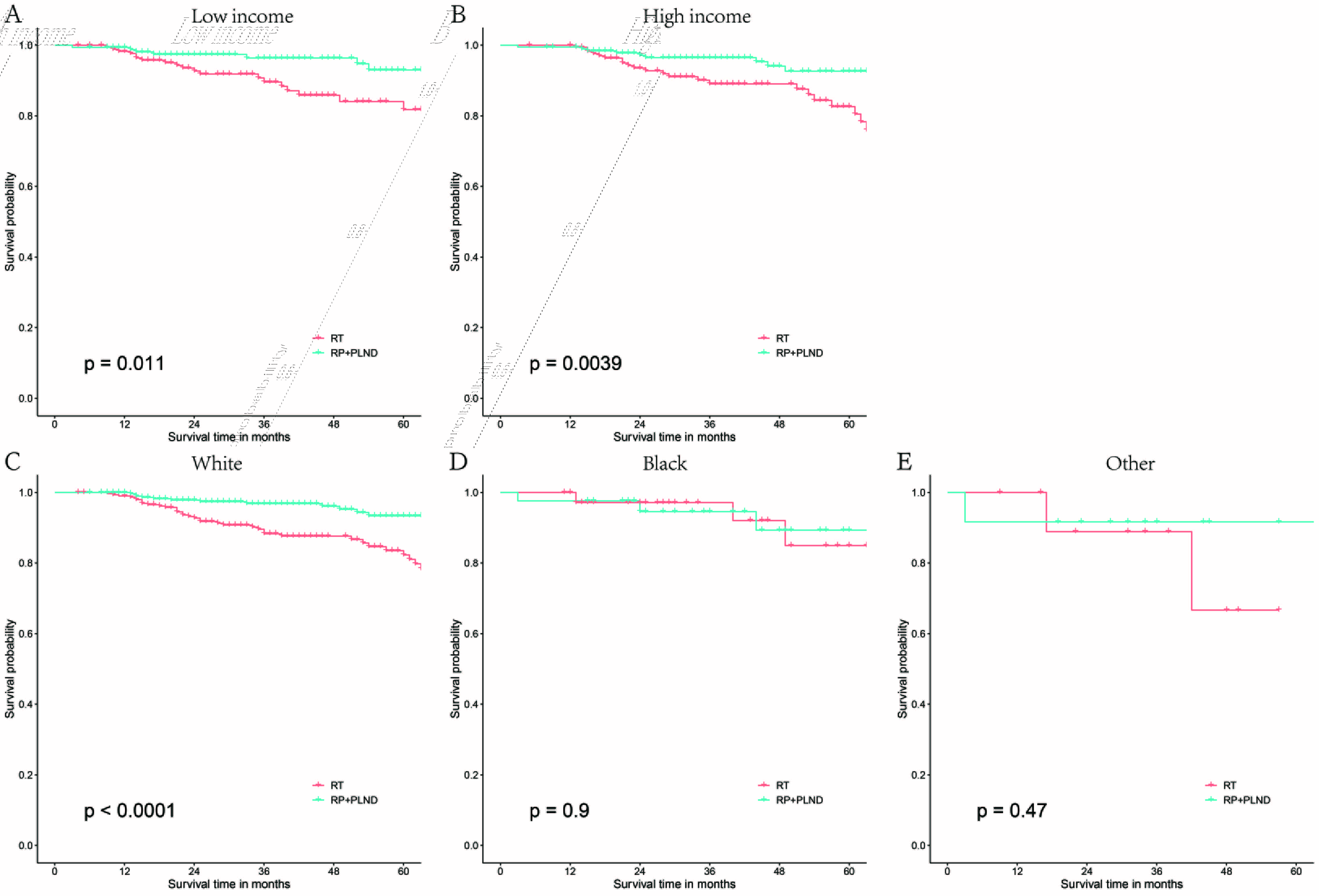

Supplement: Supplementary Figure 3 — Kaplan-Meier curves of OS for cN1M0 prostate cancer patients treated with RP+PLND versus radiation therapy, stratified by different variables: household income (A, B) and race (C–E). OS, overall survival; RP, radical prostatectomy; PLND, pelvic lymph node dissection. [file Image_3.jpeg]

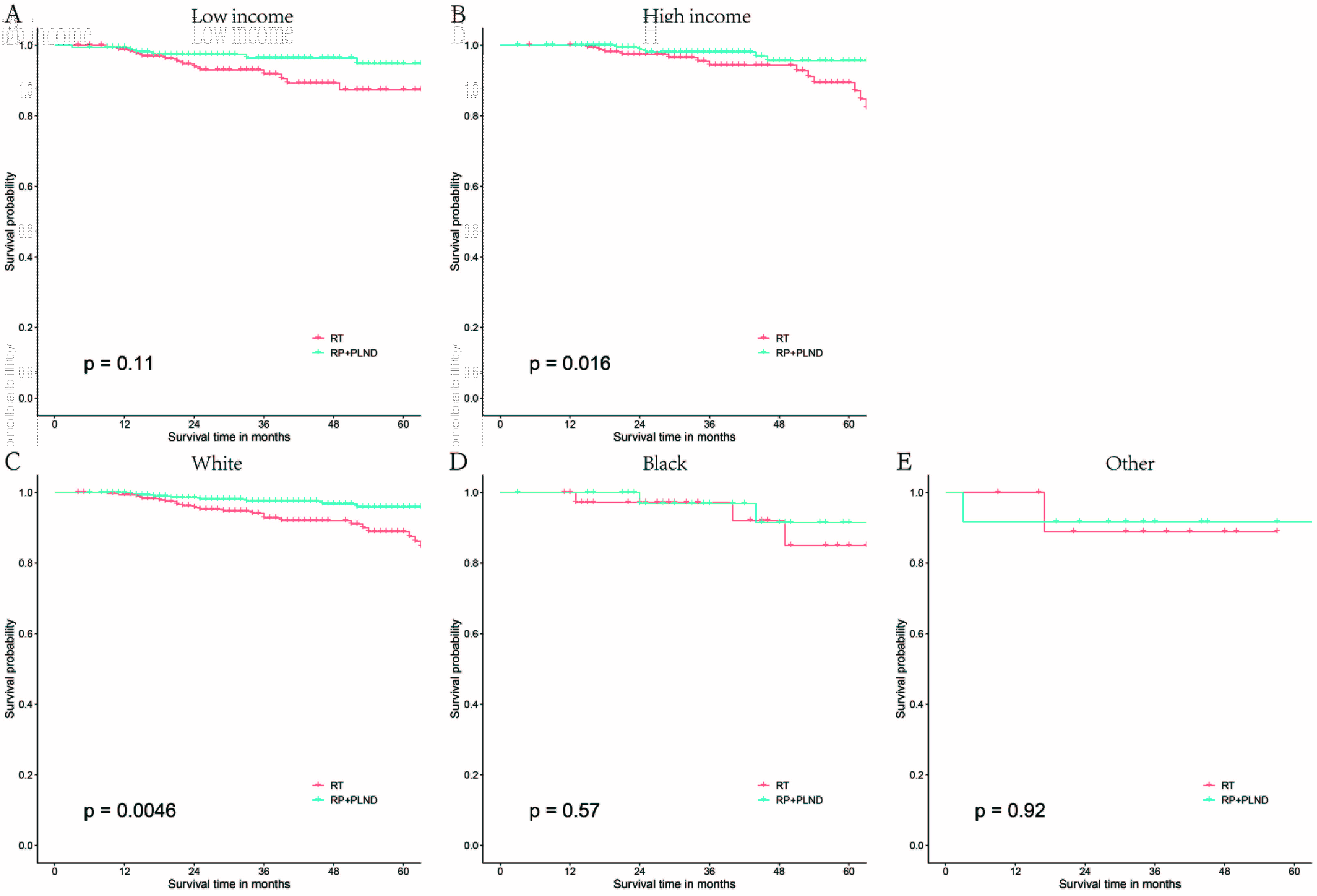

Supplement: Supplementary Figure 4 — Kaplan-Meier curves of CSS for cN1M0 prostate cancer patients treated with RP+PLND versus radiation therapy, stratified by different variables: household income (A, B) and race (C–E). CSS, cancer-specific survival; RP, radical prostatectomy; PLND, pelvic lymph node dissection. [file Image_4.jpeg]
