# Supplementary material for: Evaluation of the role of local therapy in patients with cN1M0 prostate cancer: A population-based study from the SEER database
Source: Front Oncol. 2022 Dec 5;12:1050317. doi: 10.3389/fonc.2022.1050317 (PMC9760928; doi:10.3389/fonc.2022.1050317)
Supplement: Supplementary file 5 [file Table_1.doc]

Table S1: Basic characteristics of cN1M0 prostate cancer patients, stratified by the administration of local therapy

|  | Total | No Local therapy | Local therapy | P* |
| --- | --- | --- | --- | --- |
| Variable |  |  |  |  |
| N | 2,590 | 371 | 2,219 |  |
| Age |  |  |  | <0.001 |
| <60 | 732 (28.26) | 76 (20.49) | 656 (29.56) |  |
| [60-75) | 1,590 (61.39) | 201 (54.18) | 1,389 (62.60) |  |
| ≥75 | 268 (10.35) | 94 (25.34) | 174 (7.84) |  |
| Median (IQR), y | 64.00 (59.00-69.00) | 68.00 (62.00-75.00) | 64.00 (58.00-68.00) | <0.001 |
| Race |  |  |  | 0.175 |
| White | 2,094 (80.85) | 287 (77.36) | 1,807 (81.43) |  |
| Black | 366 (14.13) | 61 (16.44) | 305 (13.74) |  |
| Other | 130 (5.02) | 23 (6.20) | 107 (4.82) |  |
| Clinical T stage |  |  |  | <0.001 |
| T1 | 1,162 (44.86) | 145 (39.08) | 1,017 (45.83) |  |
| T2 | 900 (34.75) | 119 (32.08) | 781 (35.20) |  |
| T3 | 441 (17.03) | 75 (20.22) | 366 (16.49) |  |
| T4 | 87 (3.36) | 32 (8.63) | 55 (2.48) |  |
| PSA |  |  |  | <0.001 |
| <4 | 117 (4.52) | 11 (2.96) | 106 (4.78) |  |
| [4-10) | 952 (36.76) | 69 (18.60) | 883 (39.79) |  |
| [10-20) | 731 (28.22) | 107 (28.84) | 624 (28.12) |  |
| ≥20 | 790 (30.50) | 184 (49.60) | 606 (27.31) | <0.001 |
| Median (IQR), ng/mL | 11.95 (7.00-23.70) | 19.80 (41.70-10.70) | 11.00 (21.10-6.80) | <0.001 |
| ISUP grade group |  |  |  |  |
| ISUP 1 | 108 (4.17) | 14 (3.77) | 94 (4.24) |  |
| ISUP 2 | 899 (34.71) | 65 (17.52) | 834 (37.58) |  |
| ISUP 3 | 638 (24.63) | 86 (23.18) | 552 (24.88) |  |
| ISUP 4 | 836 (32.28) | 168 (45.28) | 668 (30.10) |  |
| ISUP 5 | 109 (4.21) | 38 (10.24) | 71 (3.20) |  |
| Household income |  |  |  | 0.039 |
| Low | 1,509 (58.26) | 198 (53.37) | 1,311 (59.08) |  |
| High | 1,081 (41.74) | 173 (46.63) | 908 (40.92) |  |

Data were n (%), unless otherwise specified. IQR: interquartile range; PSA: prostate specific antigen; ISUP: International Society of Urological Pathology;

Median household income: defined by earnings above the median of the median household income in this sample

*P: Comparisons between patients with and without local therapy
