# Supplementary material for: Evaluation of the role of local therapy in patients with cN1M0 prostate cancer: A population-based study from the SEER database
Source: Front Oncol. 2022 Dec 5;12:1050317. doi: 10.3389/fonc.2022.1050317 (PMC9760928; doi:10.3389/fonc.2022.1050317)
Supplement: Supplementary file 6 [file Table_2.doc]

Table S2: Basic characteristics of cN1M0 prostate cancer patients, stratified by the administration of local therapy (After PSM)

|  | No Local therapy | Local therapy | P* |
| --- | --- | --- | --- |
| Variable |  |  |  |
| N | 371 | 371 |  |
| Age |  |  | 0.082 |
| <60 | 76 (20.49) | 62 (16.71) |  |
| [60-75) | 201 (54.18) | 231 (62.26) |  |
| ≥75 | 94 (25.34) | 78 (21.02) |  |
| Race |  |  | 0.560 |
| White | 287 (77.36) | 294 (79.25) |  |
| Black | 61 (16.44) | 51 (13.75) |  |
| Other | 23 (6.20) | 26 (7.01) |  |
| Clinical T stage |  |  | 0.296 |
| T1 | 145 (39.08) | 130 (35.04) |  |
| T2 | 119 (32.08) | 133 (35.85) |  |
| T3 | 75 (20.22) | 85 (22.91) |  |
| T4 | 32 (8.63) | 23 (6.20) |  |
| PSA |  |  | 0.737 |
| <4 | 11 (2.96) | 10 (2.70) |  |
| [4-10) | 69 (18.60) | 73 (19.68) |  |
| [10-20) | 107 (28.84) | 94 (25.34) |  |
| ≥20 | 184 (49.60) | 194 (52.29) |  |
| ISUP grade group |  |  | 0.820 |
| ISUP 1 | 14 (3.77) | 11 (2.96) |  |
| ISUP 2 | 65 (17.52) | 76 (20.49) |  |
| ISUP 3 | 86 (23.18) | 79 (21.29) |  |
| ISUP 4 | 168 (45.28) | 166 (44.74) |  |
| ISUP 5 | 38 (10.24) | 39 (10.51) |  |
| Household income |  |  | 0.268 |
| Low | 198 (53.37) | 213 (57.41) |  |
| High | 173 (46.63) | 158 (42.59) |  |

Data were n (%), unless otherwise specified. PSM: propensity score matching; IQR: interquartile range; PSA: prostate specific antigen; ISUP: International Society of Urological Pathology;

Median household income: defined by earnings above the median of the median household income in this sample

*P: Comparisons between patients with and without local therapy
