# Supplementary material for: Evaluation of the role of local therapy in patients with cN1M0 prostate cancer: A population-based study from the SEER database
Source: Front Oncol. 2022 Dec 5;12:1050317. doi: 10.3389/fonc.2022.1050317 (PMC9760928; doi:10.3389/fonc.2022.1050317)
Supplement: Supplementary file 7 [file Table_3.doc]

Table S3: Basic characteristics of cN1M0 prostate cancer patients treated with local therapy, RT vs. RP+PLND

|  | RT | RP+PLND | P* |
| --- | --- | --- | --- |
| Variable |  |  |  |
| N | 544 | 1,675 |  |
| Age |  |  | <0.001 |
| <60 | 88 (16.18) | 568 (33.91) |  |
| [60-75) | 348 (63.97) | 1,041 (62.15) |  |
| ≥75 | 108 (19.85) | 66 (3.94) |  |
| Median (IQR), y | 67.00 (62.00-73.00) | 63.00 (58.00-67.00) | <0.001 |
| Race |  |  | 0.292 |
| White | 436 (80.15) | 1,371 (81.85) |  |
| Black | 75 (13.79) | 230 (13.73) |  |
| Other | 33 (6.07) | 74 (4.42) |  |
| Clinical T stage |  |  | <0.001 |
| T1 | 161 (29.60) | 856 (51.10) |  |
| T2 | 165 (30.33) | 616 (36.78) |  |
| T3 | 177 (32.54) | 189 (11.28) |  |
| T4 | 41 (7.54) | 14 (0.84) |  |
| PSA |  |  | <0.001 |
| <4 | 25 (4.60) | 81 (4.84) |  |
| [4-10) | 149 (27.39) | 734 (43.82) |  |
| [10-20) | 124 (22.79) | 500 (29.85) |  |
| ≥20 | 246 (45.22) | 360 (21.49) |  |
| Median (IQR), ng/mL | 17.600 (8.150-34.375) | 10.200 (6.500-17.700) | <0.001 |
| ISUP grade group |  |  | <0.001 |
| ISUP 1 | 12 (2.21) | 82 (4.90) |  |
| ISUP 2 | 111 (20.40) | 723 (43.16) |  |
| ISUP 3 | 156 (28.68) | 396 (23.64) |  |
| ISUP 4 | 215 (39.52) | 453 (27.04) |  |
| ISUP 5 | 50 (9.19) | 21 (1.25) |  |
| Household income |  |  | <0.001 |
| Low | 266 (48.90) | 1,045 (62.39) |  |
| High | 278 (51.10) | 630 (37.61) |  |

Data were n (%), unless otherwise specified. RT: radiotherapy; RP: radical prostatectomy; PLND: pelvic lymph node dissection; IQR: interquartile range; PSA: prostate specific antigen; ISUP: International Society of Urological Pathology;

Median household income: defined by earnings above the median of the median household income in this sample

*P: Comparisons between patients treated with RT and RP+PLND
