# Supplementary material for: Evaluation of the role of local therapy in patients with cN1M0 prostate cancer: A population-based study from the SEER database
Source: Front Oncol. 2022 Dec 5;12:1050317. doi: 10.3389/fonc.2022.1050317 (PMC9760928; doi:10.3389/fonc.2022.1050317)
Supplement: Supplementary file 8 [file Table_4.doc]

Table S4: Basic characteristics of cN1M0 prostate cancer patients treated with local therapy, RT vs. RP+PLND (After PSM)

|  | RT | RP+PLND | P value |
| --- | --- | --- | --- |
| Variable |  |  |  |
| N | 356 | 356 |  |
| Age |  |  | 0.401 |
| <60 | 66 (18.54) | 62 (17.42) |  |
| [60-75) | 247 (69.38) | 261 (73.31) |  |
| ≥75 | 43 (12.08) | 33 (9.27) |  |
| Race |  |  | 0.867 |
| White | 307 (86.24) | 302 (84.83) |  |
| Black | 38 (10.67) | 42 (11.80) |  |
| Other | 11 (3.09) | 12 (3.37) |  |
| Clinical T stage |  |  | 0.659 |
| T1 | 137 (38.48) | 130 (36.52) |  |
| T2 | 125 (35.11) | 140 (39.33) |  |
| T3 | 89 (25.00) | 80 (22.47) |  |
| T4 | 5 (1.40) | 6 (1.69) |  |
| PSA |  |  | 0.314 |
| <4 | 15 (4.21) | 10 (2.81) |  |
| [4-10) | 118 (33.15) | 129 (36.24) |  |
| [10-20) | 91 (25.56) | 103 (28.93) |  |
| ≥20 | 132 (37.08) | 114 (32.02) |  |
| ISUP grade group |  |  | 0.389 |
| ISUP 1 | 12 (3.37) | 4 (1.12) |  |
| ISUP 2 | 90 (25.28) | 92 (25.84) |  |
| ISUP 3 | 106 (29.78) | 108 (30.34) |  |
| ISUP 4 | 139 (39.04) | 142 (39.89) |  |
| ISUP 5 | 9 (2.53) | 10 (2.81) |  |
| Household income |  |  | 0.409 |
| Low | 176 (49.44) | 164 (46.07) |  |
| High | 180 (50.56) | 192 (53.93) |  |

Data were n (%), unless otherwise specified. PSM: propensity score matching; RT: radiotherapy; RP: radical prostatectomy; PLND: pelvic lymph node dissection; IQR: interquartile range; PSA: prostate specific antigen; ISUP: International Society of Urological Pathology;

Median household income: defined by earnings above the median of the median household income in this sample

*P: Comparisons between patients treated with RT and RP+PLND
